# Attitudes towards induced abortion among gynecologists in Kurdistan region of Iraq

**DOI:** 10.1186/s12905-023-02768-4

**Published:** 2023-11-16

**Authors:** Gashaw Khalid, Abubakir Majeed Saleh, Nazar Shabila, Malin Bogren, Delér Shakely

**Affiliations:** 1 Institute of Medicine, University of Gothenburg, Gothenburg, Sweden; 2 College of Medicine, Hawler Medical University, Erbil, Iraq; 3 Department of Nursing, Faculty of Nursing, Tishk International University, Erbil, Iraq; 4 College of Health Sciences, Catholic University in Erbil, Erbil, Iraq; 5 Institute of Health and Care Sciences, University of Gothenburg, Gothenburg, Sweden; 6 Department of Public Health and Community Medicine, Institute of Medicine, University of Gothenburg, Gothenburg, Sweden

**Keywords:** Abortion, Legalization, Attitudes, Middle East Region, Reproductive health, Healthcare policies, Medical ethics

## Abstract

**Background:**

Unsafe abortion is a major contributor to maternal morbidity and mortality in countries where induced abortion is restricted. In Kurdistan Region of Iraq, induced abortion is strictly forbidden except for life-threatening situations, increasing the risk of seeking unsafe abortions. Attitudes among healthcare professionals who directly encounter cases and consequences of induced abortion can be studied to improve women’s access to safe abortion. This study aimed to examine attitudes towards induced abortion among gynecologists in Kurdistan Region of Iraq.

**Methods:**

This cross-sectional facility-based study was conducted in the first quarter of 2022 in Kurdistan Region of Iraq in the cities of Erbil, Sulaymaniyah, and Duhok. Convenient sampling was used to invite 330 gynecologists to participate, with 171 ultimately completing the questionnaire, giving a response rate of 53%. Questionaries using the Taylor and Whitehead abortion attitude scale were sent in person or digitally. The data was then analyzed using Chi-square and Fisher’s exact tests to determine the independence of attitudes and associations between attitudes and sociodemographic factors.

**Results:**

Among 171 gynecologists, 25% of the gynecologists agreed that induced abortion is unacceptable under any circumstances. Most (71%) disagreed that a woman has the right to choose to have an induced abortion. Around 51% considered induced abortion murder, and 41% agreed that induced abortion goes against all morals. Around 57% disagreed with the legalization of induced abortion, while 43% agreed. Gynecologists who were unmarried (P = 0.025), under the age of 40 (P = 0.044), and with less than 10 years of clinical experience (P = 0.043) were more likely to support the legalization of induced abortion in Kurdistan Region. None of the variables was found to be independently associated with attitudes towards abortion legalization.

**Conclusions:**

Despite some younger gynecologists having more favorable attitudes towards induced abortion, most gynecologists in Kurdistan Region had less favorable views. Most gynecologists were willing to provide post-abortion care regardless of their legal status. We recommend conducting more studies to investigate the consequences of current abortion legislation among women in need of induced abortion in Kurdistan Region of Iraq.

**Supplementary Information:**

The online version contains supplementary material available at 10.1186/s12905-023-02768-4.

## Introduction

Access to safe and legal abortion is a critical component of women’s sexual and reproductive health and rights, yet some countries restrict women’s access to this essential healthcare service. Despite these restrictions, induced abortion, referring to the termination of pregnancy using drugs or surgical intervention, remains a common medical procedure worldwide. Evidence shows that limiting access to safe abortion services leads to increased unsafe abortions, which can result in maternal morbidity and mortality [1].

According to the World Health Organization (WHO) estimations, nearly 45% of all abortions are categorized as unsafe [1]. The WHO defines unsafe abortions as procedures performed under conditions of inadequate information, lacking approval, or within environments that fail to adhere to medical standards [2]. This pressing issue underscores the urgent necessity to address restricted abortion access and its potential repercussions on global public health [3].

Unintended pregnancies resulting from inadequate access to modern contraception are a leading cause of induced abortions, a substantial proportion of which are unsafe [1]. Unsafe abortions account for 4.7–13.2% of maternal fatalities annually [4]. According to estimates, 30 women in developed nations pass away for every 100,000 unsafe abortions. This rate increases to 220 deaths per 100,000 unsafe abortions in underdeveloped countries [1].

The performance of abortions is expressly prohibited by the main principles of the criminal law of Iraq, as stated in Article 417 (section four) of the Penal Code. However, it leaves room for several exceptions in dire circumstances. To protect the life and health of the expectant mother and due to fetal abnormalities, induced abortion is permitted in Iraq. The law in Iraq also permits abortions in rape and incest instances [5]. Despite the prohibition on abortion in Kurdistan Region, a study of 7551 married women aged 15–49 years from three governorates of Kurdistan Region showed that approximately 28% had undergone an induced abortion [6], indicating that unsafe abortions are being performed.

Women must be able to use contraception to preserve their health and rights. In Iraq and Kurdistan Region, the government’s health institutes provide free family planning services, and contraceptives are accessible in most pharmacies [7]. According to the WHO data, contraception is used by 53% of Iraqi women [8]. In Kurdistan Region, more women use contraception. However, one study showed that the unmet contraceptive needs were around 28% [9].

The health care system in Iraq has historically adopted a hospital-oriented and capital-intensive model that depends on large scales of medicines and medical equipment. In the public sector, health services are provided through a network of primary healthcare centers and hospitals where services are provided at low charges to all people with an equal chance for access. However, this causes overuse of health services and overcrowding of health facilities. Over the last decade, Iraq and Kurdistan Region have witnessed a rapid expansion of a largely unregulated private sector. The private sector primarily depends on out-of-pocket payments with no existing health insurance system [10, 11].

Views, stigma, and accessibility concerning abortion can be influenced by culture and community. Within contemporary psychology, an individual’s attitude towards an action is typically still determined by whether they view the activity as positive or negative [12]. The impact of culture and community on attitudes and perceptions of abortion in the Kurdistan Region of Iraq has not yet been studied. However, a study among Asian Americans shows how cultural perspectives on sexual and reproductive health attitudes might affect both the social acceptance of abortion and the experiences of obtaining abortion treatment. Participants discussed how religion has negatively impacted their family’s and community’s attitudes against abortion. The majority of participants chose not to tell their families about their abortions, which led to stigma and made participants feel alone throughout their abortion experience [13].

While knowledge of the legal framework surrounding induced abortion in Kurdistan Region is apparent, there is little research about the opinions and perspectives of experts in the field and very few from nearby countries (one from medical students in Jordan, and one among gynecologists in one Egyptian city). Understanding this is essential, as policymakers should be aware of the viewpoints of experts who are directly involved in performing induced abortions, and a change in viewpoints could have practical repercussions for the provision of induced abortion in the future. Therefore, the present study aims to examine the attitudes towards induced abortion among gynecologists in Kurdistan Region of Iraq and the contributing factors (gender, ethnicity, religion, marital status) in determining these attitudes.

## Methods

### Design and setting of the study

A cross-sectional facility-based study was conducted between March and April 2022 in Kurdistan Region of Iraq using a validated questionnaire [14].

### Study participants

Convenience sampling was used, and the sample size of 201 gynecologists was estimated through Epi info software. The population of gynecologists in Kurdistan Region is approximately 420, and we had an expected frequency of 50%, with a margin of error of 5%, design effect 1. 330 gynecologists were invited to participate in the study, either in person or digitally, with 171 ultimately completing the questionnaire. Participants worked in both the private and public sectors, resulting in an overall response rate of 53%. As dual practice is permitted in Iraq and Kurdistan Region, most gynecologists work in both the public sector during morning hours and the private sector in the afternoon. Therefore, depending on availability and convenience, gynecologists were approached in either the public or private sector. Most participants worked in major maternity hospitals located in urban centers, with some working in primary healthcare centers. Female gynecologists predominated, and male gynecologists were relatively rare in Kurdistan Region.

### Questionnaire

The questionnaire included three broad sections: section (i) sociodemographic characteristics regarding age, gender, marital status, years of practice, ethnicity, religion, and if the participants consider themselves religious or secular; section (ii) used the validated Taylor and Whitehead abortion attitude consisting of 10-item questionnaire with four-point Likert scale (strongly agree, agree, disagree, strongly disagree) [14] regarding the gynecologists’ attitudes towards abortion; section (iii) concerned the participants encounter, practices and attitudes regarding induced abortion and abortion complications.

According to Taylor & Whitehead (2014), a homogeneity Cronbach´s Alpha test shows a reliability of α = 0.92. Concurrent validity was revealed when compared to another scale (Hess and Rueb) measuring abortion attitude: r (92) = − 0.81, p < 0.01). The lack of a neutral point and the ease of construction of this scale are further benefits [14].

### Data collection

In Erbil, 180 specialists in gynecology and postgraduate student doctors doing their clinical practice in gynecology were approached. The data was collected from Erbil Maternal Hospital and two private clinics in Erbil and questionnaires were sent digitally to gynecologists. 150 online invitations were sent to gynecologists in Sulaymaniyah and Duhok of whom 30 gynecologists participated.

Written or online written consent was obtained from all participants who participated. The questionnaire was self-administered and handed out in envelopes together with the consent form by the first author personally to each participant and returned to the author in the same format. Regarding digital participation, the questionnaires had to be filled in analogically, and the responses had to be scanned and sent back digitally for participation to be considered.

### Missing data

Responses with significant missing data were excluded from the analysis. Initially, 174 engaged in the study, but due to incomplete answers, three participants were excluded, giving the sample size of 171 gynecologists. Section 3 of the questionnaire was not completed by five of the participants. This could be explained by their unwillingness to respond because of the question’s sensitivity, a lack of understanding, or other unidentified causes.

### Statistical analysis

The collected data were processed using the IBM SPSS ® 26 Statistics software. The Pearson Chi-square test was used to compare categorical data and evaluate the factors influencing the knowledge and attitudes among study participants. Fisher’s exact test was used when more than 20% of cells had expected frequencies less than 5. Bonferroni correction was used to adjust for Type I errors that might have resulted from multiple comparisons across several variables. The results were compared between demographic groupings as well as between different items in the analysis.

Multivariable logistic regression was used to control for confounding factors and determine the factors independently associated with the outcome. Initially, variables were considered for inclusion in the multivariate logistic regression model based on a significant univariate test with a p-value cut-off point of 0.25 considered the threshold for inclusion. However, this resulted in having only three variables for inclusion (age, marital status, and year of experience). Thus, it was decided to include all the variables in the multivariable logistic regression as all of them were considered important variables that could affect the results. However, the sex variable was excluded due to having a very small number of male participants (only 2). For the logistic regression, it was assumed that the dependent and independent variables were linearly related. The Box-Tidwell test was used to check if this assumption applied to our dataset. Adjusted Odds ratios (ORs) and 95% confidence intervals were calculated. The odds ratio is the ratio of the odds of the event happening in one group compared to another group. An adjusted odds ratio is an odds ratio that controls for other predictor variables in a model as it assesses the dynamics between the predictors. P-values < 0.05 were considered statistically significant.

### Ethical considerations

All participants who engaged in the study gave their consent, and the questionnaires and consent forms were personally given to each participant. Filling out the consent form was fully voluntary. Verbal informed permission was gained from participants who wanted to remain anonymous and did not want to sign any paperwork. The study’s goal, the participants’ anonymity, and the study’s complete voluntariness were all explained in the consent form, which was provided on a separate sheet. Additionally, participants were told that they could opt out of the study at any time, and contact information for the researchers was provided in case of any questions.

The Hawler Medical University’s Research Ethics Committee in Erbil, Iraq’s Kurdistan Region, granted their clearance (registration number SC.E.C. 4). The Helsinki Declaration’s guiding principles were used to perform the study. Participants in the study provided written, fully informed permission. The information was kept private, and it was anonymous.

### Result

#### Study population

Of 330 gynecologists invited, 171 completed the questionnaire. 125 (73%) participants were from Erbil and 46 (27%) from Sulaymaniyah and Duhok. The mean age was 37 years, and only two participants were male. Among the participants, 83% were married, and the result showed religious and ethnic diversity among the gynecologists in Kurdistan Region of Iraq, with the majority being Kurdish (90%) and Muslim (97%) (Table 1). The mean duration of clinical practice post-graduation was 10 years. The sociodemographic factors of the participants are presented in Table 1.

**Table 1:** The sociodemographic factors of the study population

Characteristics	n n = 171	% (95% CI)
Sex		
Women	169	98.8 (97.2–100)
Men	2	1.2 (0.0-2.8)
**Age**		
20–29	28	16.4 (10.9–21.9)
30–39	90	52.6 (45.1–60.1)
40–49	40	23.4 (17.1–29.7)
≥ 50	12	7.0 (3.2–10.8)
Mean +- SD	37 ± 8	
**Marital status**		
Married	141	81.5 (75.7–87.3)
Single	27	16.8 (11.2–22.4)
Divorced	3	1.7 (0.0-3.6)
**Years of practice**		
0–9	113	66.1 (59.0-73.2)
10–19	45	26.3 (19.7–32.9)
20–29	7	4.1 (1.1–7.1)
≥ 30	6	3.5 (0.7–6.3)
Mean +- SD	10 ± 8	
**Ethnicity**		
Kurdish	153	89.5 (84.9–94.1)
Arabic	13	7.6 (3.6–11.6)
Turkman	3	1.8 (0.0-3.8)
Assyrian	2	1.2 (0.0-2.8)
**Religion**		
Muslim	165	96.5 (93.7–99.3)
Christian	5	2.9 (0.4–5.4)
Yazidi	1	0.6 (0.0-1.8)
**Religious belief**		
Religious	156	91.2 (87.0-95.4)
Secular	15	8.8 (4.6–13.0)

### Attitudes

**Fig. 1:**
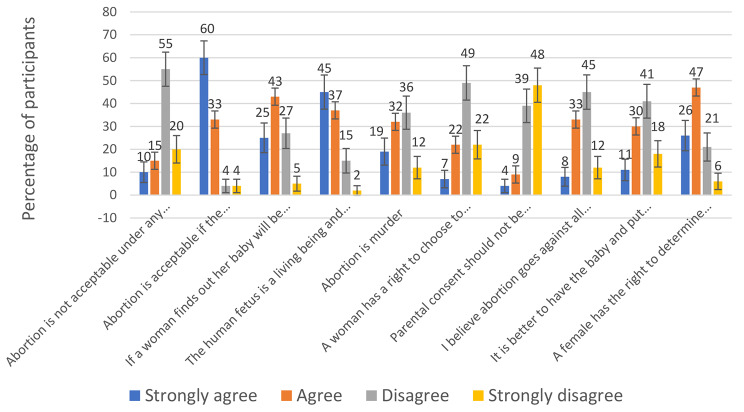
Attitudes towards induced abortion among gynecologists in Kurdistan Region of Iraq. Taylor and Whitehead abortion attitude scale consisting of a 10-item questionnaire with a four-point Likert scale (1- strongly agree, 2- agree, 3- disagree, 4- strongly disagree)

Among the gynecologists, 25% agreed that induced abortion is unacceptable under any circumstances, while 93% of the participants agreed that induced abortion is acceptable if the mother’s health is endangered. Regarding if a woman finds out her baby will be born with a defect and therefore has the right to abort the child, 68% agreed, and 32% disagreed. 82% of the gynecologists agreed that the human fetus is a living being and, therefore, should be protected by law and 18% disagreed. Most participants (71%) disagreed that a woman has the right to choose to have an induced abortion, and 87% believed that parental consent should be required for an abortion to be performed. Nearly half (51%) considered induced abortion murder, and 41% of the participants agreed that induced abortion goes against all morals and that it is better to have the baby and put it up for adoption than induced abortion. On the other hand, a majority (73%) agreed that a woman has the right to determine the best course for the life of her fetus, depending on the circumstances of conception. Details of the gynecologists’ attitudes towards induced abortion are shown in Fig. 1.

### Encounterments in daily work

Gynecologists in Kurdistan Region of Iraq encounter unwanted pregnancies, induced abortion, complications of induced abortion, self-induced abortion, and complications of self-induced abortion in their daily work. However, these encounters are met to varying degrees.

**Fig. 2:**
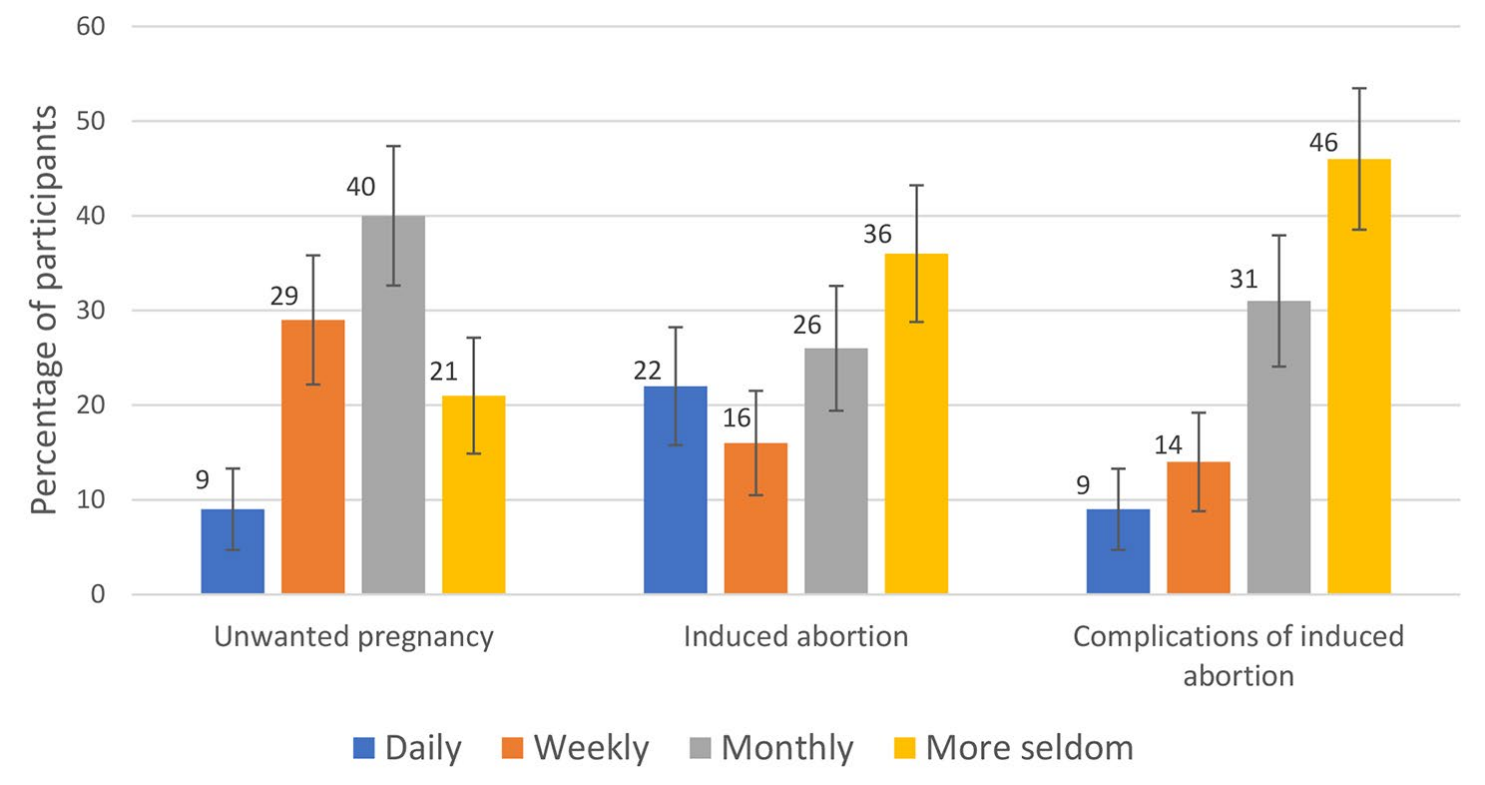
Encounters in daily work among gynecologists in Kurdistan Region of Iraq

9% of the physicians meet patients with unwanted pregnancies daily, 29% weekly, 40% monthly, and 21% more seldom. 22% of physicians meet patients who have had induced abortions daily, 16% meet induced abortion weekly, and 26% monthly. Complications of induced abortion are seen daily among 9% of the physicians, 14% weekly, and 31% monthly (Fig. 2).

### Encounterments of Complications of induced abortion

**Fig. 3:**
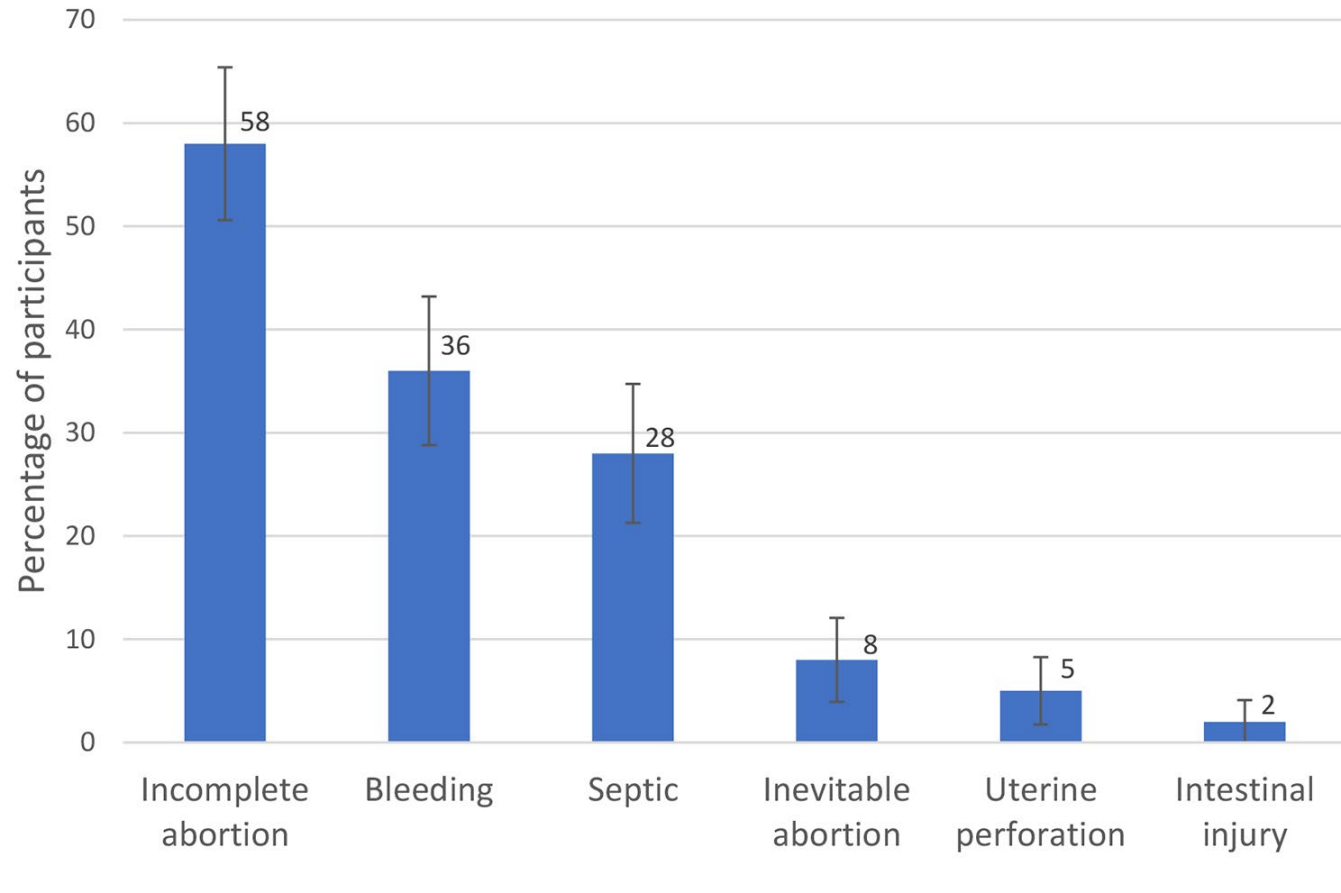
Complications of induced abortion as encountered by gynecologists

The most common complication following induced abortion seen among more than half of the gynecologists in Kurdistan Region of Iraq was incomplete abortion (58%), followed by bleeding (36%). Septic complications following an induced abortion were encountered among 28% of the gynecologists. Other complications seen were Inevitable abortion (8%), Uterine perforation (5%), and intestinal injury (2%). 97% of the physicians participating in the study offered post-abortion care to patients who sought medical help for abortion-related complications (Fig. 3).

### Legalization of induced abortion in Kurdistan Region

Concerning the attitude towards abortion legalization in Kurdistan Region of Iraq, 43.4% agreed that induced abortion should be legalized, while 56.6% disagreed. Age groups, marital status, and years of practice all showed statistical significance. Gynecologists under 40 were more in favor of legalizing induced abortion than those above that age (48.7% vs. 32.1%, P = 0.044). Similarly, gynecologists with less than 10 years of experience in the clinical setting held more favorable opinions on the legalization of induced abortion in Kurdistan Region of Iraq than those with more than 10 years of experience (49.1% vs. 32.8%, P = 0.043). Unmarried gynecologists also held significantly (P = 0.025) higher pro-abortion attitudes (62.1%) than those who were married (39.4%) (Table 2).

**Table 2:** Legalization of induced abortion in Kurdistan Region and participants sociodemographic factors

Characteristic N = 166	Attitude towards legalization of abortion				P-value	
	Agree		Disagree			
	N	% (95% CI)	N	% (95% CI)		
**Total**	72	43.4 (35.8–50.9)	94	56.6 (49.1–64.2)		
**Sex**						
Women	72	43.9 (36.3–51.5)	92	56.1 (48.5–63.7)	0.506*	
Men	0	0 (0.0–0.0)	2	100 (100.0-100.0)		
**Age-groups**						
< 40	55	48.7 (39.5–57.9)	58	51.3 (42.1–60.5)	0.044	
≥ 40	17	32.1 (19.5–44.6)	36	67.9 (55.4–80.5)		
**Marital status**						
Ever married	54	39.4 (31.2–47.6)	83	60.6 (52.4–68.8)	0.025	
Not married	18	62.1 (44.4–79.7)	11	37.9 (20.3–55.6)		
**Years of practice**						
0–10	53	49.1 (39.6–58.5)	55	50.9 (41.5–60.4)	0.043	
> 10	19	32.8 (20.7–44.8)	39	67.2 (55.2–79.3)		
**Ethnicity**						
Kurdish	63	42.6 (34.6–50.5)	85	57.4 (49.5–65.4)	0.548	
Other	9	50.0 (26.9–73.1)	9	50.0 (26.9–73.1)		
**Religion**						
Muslim	68	42.5 (34.8–50.2)	92	57.5 (49.8–65.2)	0.405*	
Other	4	66.7 (28.9–100)	2	33.3 (0.0-71.1)		
**Are you religious or secular?**						
Religious	64	42.4 (34.5–50.3)	87	57.6 (49.7–65.5)	0.414	
Secular	8	53.3 (28.1–78.6)	7	46.7 (21.4–71.9)		

*Fisher’s exact test

After adjusting for confounding factors through multivariable logistic regression, none of the variables was found to be independently associated with attitudes towards abortion legalization (Table 3). This indicates that each of the age groups, marital status, and years of practice variables that were significantly associated with attitude towards the legalization of abortion did not work independently, but the impact of each one was dependent on the impact of other variables.

**Table 3:** Multivariable logistic regression analysis of the variables associated with the attitude towards legalization of abortion in Kurdistan Region

Characteristic	Beta	Standard error	P-value	Odds ratio	95% Confidence interval for odds ratio	
					Lower	Upper
Age (< 40)	0.371	0.624	0.552	1.4	0.4	4.9
Marital status (Single)	0.647	0.466	0.165	1.9	0.8	4.8
Years of experience (0–10 years)	0.316	0.603	0.600	1.4	0.4	4.5
Ethnicity (Others)	0.135	0.599	0.822	1.1	0.4	3.7
Religion (Others)	0.605	1.047	0.563	1.8	0.2	14.3
Religious (Secular)	0.189	0.571	0.741	1.2	0.4	3.7

## Discussion

The study conducted on gynecologists’ attitudes towards induced abortion in the Kurdistan Region of Iraq revealed that younger gynecologists tended to have more favorable views on induced abortion. Given their daily work experience with induced and self-induced abortions and the potential complications, almost all gynecologists in the region provide post-abortion care to patients seeking medical assistance. However, more than half of the gynecologists disagreed with the notion of legalizing induced abortion.

There was a significant difference among age groups about the statement, “The human fetus is a living being and therefore should be protected by law.” The results showed that gynecologists over 40 were more likely to agree with this statement, indicating a stricter view on induced abortion and a belief that the human fetus should be protected by law. This is consistent with findings from other parts of the world, where younger gynecologists tend to have more favorable attitudes towards abortion. For instance, a study conducted in Guatemala found that Compared with older gynecologists, those under 40 years of age were 7 times more likely, and 40-49-year-olds were twice as likely to approve of medical abortion for fetal death and severe eclampsia with fetal death, respectively [15]. These findings suggest that attitudes towards induced abortion may vary depending on age and experience within the medical profession.

While most gynecologists agreed that induced abortion is acceptable if the mother’s health is endangered if the baby will be born with a defect, and that a woman can determine the best course of action for the life of the fetus, a significant proportion (71%) disagreed that a woman has the right to choose to have an abortion. This may be because gynecologists in Kurdistan only support induced abortion when there are medical indicators for the procedure. While 93% of gynecologists agree that induced abortion is allowed if the mother’s health is in danger, 25% believe induced abortion is unacceptable in all situations, indicating inconsistency.

The findings of this study indirectly show that induced abortion and self-induced abortion exist in Kurdistan Region. Gynecologists encounter women who have undergone induced abortions and the health risks that can come with it. This suggests that induced abortions occur to a greater extent than the law advocates and that this needs further exploration. Among married women in Mosul, Iraq, 13.5% had attempted to induce an abortion for birth control at some point, either through engaging in strenuous physical activity, using herbal remedies, or administering pharmaceuticals [16]. Gynecologists in Kurdistan Region treat women who have complications from induced abortion regularly. Being enlightened about the reality of the health risks these women are exposed to as a result of unsafe abortions can give gynecologists more supportive attitudes regarding the importance of providing safer and better conditions for induced abortions. The encounters question (section three) in the study did not specify whether there were complications from legal or illegal abortions; this point can be further explored in future studies to understand better the correlation between complications and whether the abortions were legal or illegal.

Attitudes towards the legalization of induced abortion varied in this study, with a majority (56.6%) of the study group in disagreement with its legalization in the Kurdistan Region, while 43.4% expressed agreement. The prevalence of less favorable attitudes towards induced abortion among gynecologists in this geographical area can be because the doctors simply do not want to be part of an illegal procedure, given that induced abortion is illegal in Kurdistan Region of Iraq. Moreover, our results are similar to those among students studying Medical and Health Sciences in both Jordan and Turkey [17, 18].

Considering abortion is illegal in the Kurdistan Region of Iraq and religiously prohibited, Kurdish gynecologists might deal with the abortion concept confidentially, limiting expressions on this subject in fear of stigma and judgment from other physicians or society. Abortion stigma is a negative connotation attached to women who opt to end a pregnancy, implying that they are less than the social ideals of womanhood [19]. This negative attitude may have an impact on abortion services’ availability, accessibility, quality, and acceptance [20]. A study from Uruguay also shows that despite the benefits of the legalization of abortion, clients and health professionals still experience abortion stigma and critical points [21].

In our study, the main reason for agreement or disagreement towards the legalization of induced abortion was religious beliefs. Studies from countries with similar abortion policies, such as Egypt, showed that most private gynecologists do not terminate unwanted pregnancies because of their religious beliefs [22]. In our study, we can see that 68% of the gynecologists against the legalization of induced abortion in Kurdistan Region stated religious beliefs as the reason. Similar to our findings, a study from Mexico showed that gender and religion were important factors contributing to certain attitudes toward elective abortion. Male gynecologists and those with strong religious convictions (mostly Catholics) revealed a less favorable attitude than females and physicians with weaker religious beliefs [23].

In this study, only two of the 171 participants were male. Given that gender may influence opinions regarding abortion, this significant gender gap in the sample may have an impact on the representation and generalizability of the study’s findings. According to a 2019 study, women globally scored higher on supportive attitudes about abortion than males [24]. In a study from Harar City, eastern Ethiopia, male healthcare providers were almost three times more likely than female healthcare providers to have a favorable attitude toward safe abortion services. Possible explanations for these findings include the fact that male providers are more pro-choice than female providers, or that male providers received pre-service training at a rate that was 5% higher than that of female providers, which may have affected males’ opinions to be more favorable than those of females [25].

Increased acceptance of abortion may result from promoting the legality of safe abortion across populations and environments. Significant access constraints include women’s lack of awareness of service availability and/or the legality of abortion on demand. The use of terminology such as “murder” and “immortal” in the context of describing abortion by politicians and health officials can influence the opinions and willingness to deliver the services of healthcare professionals. To change the attitudes of healthcare providers toward safe abortion services, providing pre-service or in-service training on safe abortion care and encouraging them to become informed about their nation’s abortion legislation are essential. The interaction between the medical professional and the pregnant patient who wants an abortion is subsequently impacted by these attitudes. Additionally, all training programs should include value clarification, which helps healthcare professionals discern between their views and their clients’ rights to safe reproductive health. This intervention has been shown to allow participants to reflect on their values and attitudes and how they are influenced by beliefs, ideals, and knowledge [26]. Legalizing abortion would encourage safe abortion practices, avoid unsafe abortions, and lower maternal fatalities and complications as a result of illegal abortions [27].

There is a need for the collaborative involvement of stakeholders in addressing this sensitive issue of induced abortion and its legalization. Potential stakeholders include lawmakers, religious leaders, women’s rights groups, health professionals, and medical managers. To ensure the collaborative involvement of all stakeholders, they need to understand the result of the current abortion restrictions, including health complications, maternal death, and unwanted pregnancies. They also need to perceive the consequences of increasing access to safe abortion, including improved health, but also overuse of abortion, marital conflict, and not taking preventive behavior. All stakeholders must be involved in all the stages and details of discussions regarding this sensitive issue. The impact of unsafe abortion on maternal death and morbidity was a key reason for Nepal’s restrictive abortion law being liberalized in 2002. Nepal was able to launch and scale up safe abortion services in a relatively short timescale because of careful, thorough preparation among a variety of multisectoral partners headed by Nepal’s Ministry of Health and Population [28]. Strategies used to access safe and legal abortion services in Kurdistan Region of Iraq include certifying healthcare facilities, equipping clinicians with the required tools, and teaching them to conduct abortions.

### Strengths and limitations

Given the sensitivity of the subject, the response rate of 53% of gynecologists approached in the Kurdistan Region of Iraq is a good number, and this may be because many gynecologists in Kurdistan are interested in the subject and the gynecologists have consulted appropriately. The study focuses on the performers of abortion (licensed gynecologists and obstetricians) and not the care recipients. It provides an interesting and important perspective on abortion as they are directly involved in performing abortions and daily treatment of women with unwanted pregnancies and abortion needs.

Using a validated questionnaire in our study increases the likelihood of assuring the reliability and excellence of a survey instrument and allows for the appropriate interpretation of survey results [29]. Convenience samples are quite susceptible to bias in research and never produce a statistically balanced selection of the population because the sample is chosen based on convenience rather than equal probability, which introduces sampling bias. The present study used a convenient sampling method to recruit participants, which limits the generalizability of the results to the (sub)population from which the sample was drawn [30]. The risk of response bias, which occurs when respondents and nonrespondents categorically differ in ways that affect the research [31], is another element that influences the generalizability of the findings of this study since there was a non-response rate of 47%. Although the response rate was higher than expected in the study, the sample size of 171 gynecologists is small, giving the study another limitation since “small samples undermine a study’s internal and external validity” [32].

The response rate from Sulaymaniyah and Duhok was lower than from Erbil, possibly because the questionnaires were not handed out face-to-face in those cities. Moreover, response rates to online surveys, such as the one used in this study, tend to be low. This has a possible influence on the outcome of the results as gynecologists in these cities may be different from those in Erbil, for example, in age, which was a factor that influenced attitudes towards abortion. There is also no description of whether women in these cities undergo more or less induced abortions, whether the gynecologists here are more involved in carrying out abortions, or whether they deal with complications more often, affecting their attitudes towards induced abortions.

As with any questionnaire study, there is a potential for misinterpretation of questions, such as those related to religious or secular beliefs or the term “induced abortion,“ which could have different meanings depending on the respondent’s location or cultural background. To mitigate this, future studies could include explanatory paragraphs in the consent form to help participants better understand the study’s purpose and definitions of key terms. Interviews could be conducted to further clarify participants’ responses and understand the underlying causes of conflicting opinions regarding induced abortion. In addition, exploring attitudes towards induced abortion among other study populations (midwives, married women, etc.) would be valuable to better present the attitudes among the population of Kurdistan Region of Iraq.

## Conclusions

In conclusion, the attitude towards induced abortion among gynecologists in Kurdistan Region is shown to be less favorable, although younger gynecologists had an overall more favorable viewpoint. Despite less favorable views on induced abortion, there was a willingness to provide care to women who had undergone unsafe and illegal abortions. Age, gender, religious background, local legislation, culture, and stigma are just a few of the variables that may affect gynecologists’ opinions on induced abortion. Given the unfavorable views on induced abortion, women may have detrimental health effects as a result, since unsafe abortion places a significant burden on mortality and morbidity. To improve abortion care, we advise educating healthcare professionals about the nation’s abortion regulations and providing pre-service or in-service training on safe abortion care.

### Electronic supplementary material

Below is the link to the electronic supplementary material.

Supplementary Material 1

## Data Availability

The datasets generated and/or analyzed during the current study are not publicly available due to the confidentiality required by our ethics approval but are available from the corresponding author upon reasonable request.
