# Supplementary material for: Attitudes towards induced abortion among gynecologists in Kurdistan region of Iraq
Source: BMC Womens Health. 2023 Nov 16;23:609. doi: 10.1186/s12905-023-02768-4 (PMC10655302; doi:10.1186/s12905-023-02768-4)
Supplement: Supplementary file 1 — Supplementary Material 1 [file 12905_2023_2768_MOESM1_ESM.docx]

**Questionnaire: Knowledge and attitudes towards abortion among gynecologists in Kurdistan region of Iraq**

| **Section 1 - Sociodemographic characteristics among participants** |
| --- |

1. **Age**
2. **Sex**

*Male*

*Female*

1. **Marital status**

*Married*

*Single*

*Divorced*

1. **Years of practice**
2. **Ethnicity**

*Kurdish*

*Arabic*

*Assyrian*

*Turkmen*

*Other (please specify)*

1. **Religion**

*Muslim*

*Christian*

*Other (please specify)*

1. **Do you consider yourself religious or secular?**

*Religious*

*Secular*

| **Section 2: Taylor and Whitehead abortion attitude scale (2014)** |
| --- |

*The following are questions about your own attitudes and opinions regarding abortion. There are no right, or wrong answers and all answers are completely anonymous. Please indicate*

*your level of agreement or disagreement with each statement.*

Strongly agree = 1

Agree = 2

Disagree = 3

Strongly disagree = 4

1. Abortion is not acceptable under any circumstances.

2. Abortion is acceptable if the mother's health is endangered.

3. If a woman finds out her baby will be born with a defect, she has the right to abort the child.

4. The human fetus is a living being and therefore should be protected by law.

5. Abortion is murder.

6. A woman has a right to choose to have an abortion.

7. Parental consent should not be required for an abortion to be performed.

8. I believe abortion goes against all morals.

9. It is better to have the baby and put it up for adoption than an abortion.

10. Depending on the circumstances of conception, a female has the right to determine the best course for the life of her fetus.

| **Section 3 - In your daily work how often do you encounter with women** |
| --- |

1. **With unwanted pregnancies**

*Daily*

*Weekly*

*Monthly*

*More seldom*

1. **Induced abortion**

*Daily*

*Weekly*

*Monthly*

*More seldom*

1. **Complications of induced abortion**

*Daily*

*Weekly*

*Monthly*

*More seldom*

1. **Self-induced abortion**

*Daily*

*Weekly*

*Monthly*

*More seldom*

1. **Complications of self-induced abortion**

*Daily*

*Weekly*

*Monthly*

*More seldom*

1. **What types of abortion complications do you encounter among these women?**

*Inevitable abortion*

*Incomplete abortion*

*Septic*

*Uterine perforation Intestinal injury*

*Bleeding*

1. **Do you offer post abortion care to women who have undergone unsafe/illegal abortion?**

*Yes*

*No*

1. **If no, please mention why?**

*It is illegal*

*It is against my principal*

*Because of the stigmatization by the society*

*Other reasons (please specify)*

1. **What is your opinion on legalization of abortion in KRG?**

*Strongly agree*

*Agree*

*Disagree*

*Strongly disagree*

1. **Why**

*Religious beliefs*

*Cultural beliefs*

*Ethical reasons*

*Other reasons (please specify)*
